# Autochtonal case of chronic, unifocal, pulmonary paracoccidioidomycosis with methotrexate use, in Salvador ‒ Brazil

**DOI:** 10.1016/j.bjid.2024.103768

**Published:** 2024-06-05

**Authors:** Priscila de Abreu Franco, Cesar Augusto de Araújo Neto, Sonia Regina Leite da Silva, João Carlos Coelho Filho, Carlos Brites, Jorge Luiz Pereira-Silva

**Affiliations:** a Hospital São Rafael, Instituto D'Or de Pesquisa e Ensino (IDOR), Salvador, BA, Brazil; b Universidade Federal da Bahia, Departamento de Medicina, Salvador, BA, Brazil; c Fundação José Silveira, Salvador, BA, Brazil

**Keywords:** Chronic pulmonary paracoccidioidomycosis, Epidemiology, Radiological findings, Immunosuppression

## Abstract

**Summary:**

We report an autochthonous case of mild unifocal chronic pulmonary paracoccidioidomycosis in a 48-year-old previously healthy woman with no history of possible environmental exposures in endemic rural areas, supposedly resulting from reactivation of a latent pulmonary focus secondary to the use of methotrexate for the control of Chikungunya arthropathy. Laboratory investigation ruled out other immunosuppression. Her only symptoms were a dry cough and chest pain. Diagnosis confirmed by needle lung biopsy. There were no abnormalities on physical examination nor evidence of central nervous system involvement. MRI of the total abdomen showed no involvement of other organs. Computed chest tomography showed a favorable evolution under the use of itraconazole (200 mg/day). Different tomographic presentations findings are highlighted when performed before and after treatment.

**Conclusions:**

PCM should be considered even in a woman without a history of consistent environmental exposure and in a non-endemic geographic area.

## Introduction

PCM is caused by two species of thermodimorphic fungi: *Paracoccidioides braziliensis* and *P. lutzi* and was originally described by Lutz in 1908.^1^ While the first is preferentially distributed in the south and southeast of Brazi, in Argentina and Paraguay, *P. lutzi* predominates in the Midwest and Amazon region (Brazil and Equador).^2^ Paracoccidioidomycosis (PCM) is the most common endemic systemic mycosis affecting non-immunocompromised hosts in Latin America, particularly in Brazil, Argentina, Colombia and Venezuela.^2^^,^^3^ There are imprecise data in Brazil on PCM incidence, with estimates ranging from 3 to 4 new cases/million to 1 to 3 new cases per 100,000 inhabitants per year. About 80% of cases in Brazil, were detected in the states of São Paulo, Paraná, Rio Grande do Sul, Goiás and Rondônia.^1^ In Latin America, cases are more frequently reported in Argentina, Colombia, Venezuela, Ecuador and Paraguay.^2^^,^^3^ Estimates of annual incidence in Brazil range from 0.71 cases to 3.70 cases per 100,000 inhabitants, with recent records of incidence of 9.4 cases per 100 thousand inhabitants in Rondônia – and in two municipalities, there are incidences close to 40 cases per 100 thousand inhabitants.^2^

## Case report

A 48-year-old white female patient, merchant, non-smoker, with no history of previous environmental exposures in rural area received a diagnosis of Chikungunya in July 2020. She used methotrexate 10‒20 mg/week, from August 2020 to July 2021 to control the joint involvement by Chikungunya.

In May 2021, she developed a dry cough and ventilatory-dependent chest pain on the right. There were no dysphonia, odynophagia, skin or upper airway lesions, lymph node enlargement, fever, hemoptysis, asthenia, anorexia, weight loss or neurological symptoms. A computed tomography angiography of the chest showed irregular pulmonary opacities with intermingled cavitations and septations, in the lower lobe of the right lung.

Physical examination showed no skin nor oral cavity lesions, no dysphonia, or lymph nodes enlargement. Thyroid were normal as well as cardiorespiratory auscultation. Abdomen without abnormalities on examination. Extremities without digital clubbing or swelling. Axillary temperature (36.5 °C), blood pressure (115 × 60 mmHg), heart rate (68 bpm), and respiratory rate (18 cpm) were in normal limits range. SpO_2_ 98% was on room air.

All laboratory tests performed (blood count, ESR, CRP, AST, ALT, bilirubin, alkaline phosphatase, total proteins and fractions, prothrombin time, urea, creatinine, urine summary, serum immunoglobulin dosage, serum protein electrophoresis) were within normal limits. Serological tests for HIV, *Aspergillus, Hystoplasma* and *Paracoccidioides* were negative. IgE for A*spergillus*, and ANCA, were non-reactive. PPD test induced an induration of 12 mm. Table 1 shows the laboratory tests results.

**Table 1:** Laboratory tests results at first medical visit.

Laboratory Test	Results
White blood cells count	7210 cells/mm^3^
Hemoglobin	13.9 g/dL
Platelets	278,000/mm^3^
ESR	
Glucose (fasting)	90
Triglycerides	76 mg/dL
Creatine phosphokinase	114 U/L
Reactive C protein	0.57 mg/dL
AST	25.3 U/L
ALT	24.7 U/L
Creatinine	0.8 mg/dL
Uric acid	3.4 mg/dL
Erythrocytes sedimentation rate	10 mm
Albumin	4.6 g/dL
Urinalysis	Normal

A Magnetic Resonance Imaging (MRI) of the entire abdomen only showed uterine leiomyomas and endometriosis.

Fiberoptic bronchoscopy with Bronchoalveolar Lavage (BAL) detected no visible abnormality. Direct research and cultures for mycobacteria and fungi, rapid molecular test for tuberculosis, galactomannan detection and search for neoplastic cells were all negative.

**Fig. 1:**
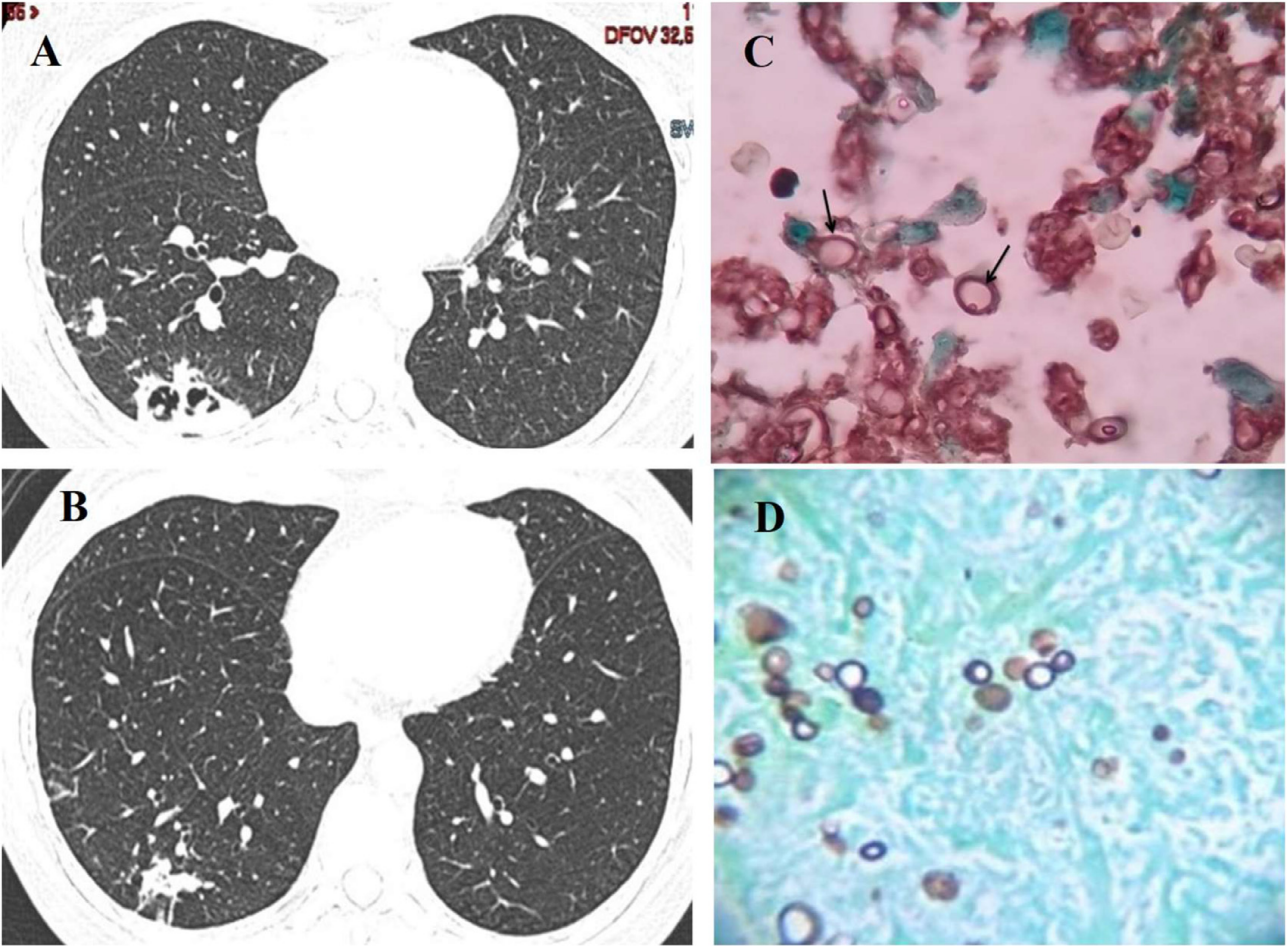
(A) Pre-treatment thorax CT showing subpleural consolidations and septations, and (B) Partial resolution of initial findings after 1 year of treatment. (C and D) Grocott staining showing levedurifom forms of *P. brasiliensis* (arrows), at 1.000 × .

A CT-guided transcutaneous needle biopsy of lung lesions was performed and typical fungal structures suggesting *P. brasiliensis* were seen at microscopy, as shown in Fig. 1C and D.

The patient received itraconazole (200 mg/day for 12 months) with complete recovery of symptoms and significant regression of CT lung lesions. Fig. 1A and B displays the radiological findings at diagnosis and after one year of treatment with itraconazole.

The patient authorized the publication of this case report, by signing an Informed Consent Form (ICF). This case report was approved by the Research Ethics Committee of Hospital São Rafael.

## Discussion

In nature, *P. brasiliensis* and *P. lutzii* develop as filamentous structures and produce infective propagules called conidia. The propagules give rise to yeast-like structures of the fungus, which will constitute its parasitic form in the tissues of the host.^4^ The great risk factor for acquiring the infection is exposure to soil contaminated with the fungus.^4^ Initially described as a primary infection affecting mostly affecting individuals engaged in agricultural activity in the first two decades of life, when they probably acquired the infection, recent evidence suggest an increasing number of atypical and severe forms, with increasing mortality rate.^5^^,^^6^ Unlike other mycoses, such as cryptococcosis, disseminated histoplasmosis, and candidiasis, PCM is not usually related to immunosuppressive diseases.^4^

Bahia is not considered an endemic region for PCM. In the municipality of Una, located in the south of the state of Bahia, in an endemic area for cutaneous leishmaniasis, 177 individuals, aged between 3 months and 73 years, were studied using intradermal tests with paracoccidioidin.^7^ Only ten individuals (5.6%) tested positive (induration > 5 mm). None of the paracoccidioidin-positive cases showed clinical evidence of the disease. Double immunodiffusion and counter immunoelectrophoresis tests were performed with the sera of the paracoccidioidin-positive individuals, with negative results for circulating anti-*P. brasiliensis*, indicating that none of the paracoccidioidin reactors had an active infectious process. The percentage of positivity obtained with paracoccidioidin, despite possible cross-reactions with histoplasmosis, suggests the occurrence of PCM, although rare, in the studied area.

In a clinical-seroepidemiological study carried out at the Hospital Estadual Universitário de Campinas, São Paulo, Brazil that included 584 individuals with PCM (492 men, 92 women, aged between 5 and 87 years), a higher incidence of the disease was detected between 41 and 50 years of age in men and between 11 and 40 years of age in women.^8^ Rural activities constituted the main occupational activity in 46% of cases. The diagnosis was confirmed by histopathological examination and detection of the fungus in smears of lesions and secretions, in sputum, or based in PCM positive serological tests, in 80% of the 584 patients studied. The expressive number of cases in this region, including 33 children under 14 years of old age characterizes the region as an important endemic area of PCM.^8^

PCM can compromise any organ or system, and chronic PCM is responsible for most cases (74–96%) of the disease.^3^^,^^4^^,^^9^ It usually presents in adults aged 30 to 60 years and predominates in males – male-to-female ratio equal to 22:1.^9^ It is known that estrogens inhibit the mycelial to yeast-like transformation of the fungus.^10^ Pulmonary involvement is present in 90% of patients, and it may be the only manifestation of the disease. In 90% of cases, lung involvement is bilateral.^11^

In addition to the lungs, the upper aerodigestive tract mucosa and skin are the sites most affected by PCM.^3^ In the present report, the only clinical findings were cough and chest pain, and diagnosis was made only by a needle lung biopsy.

In a report[12] in which 47 individuals with chronic PCM were treated with itraconazole and followed up for an average of 5.6 years, it was shown that triazole was effective in combating fungal infection but, in the long term, it was not capable of preventing the formation of fibrotic pulmonary sequelae induced by the fungus. Chronic PCM can diffusely affect the lungs, even after specific antifungal therapy.^12^ Patients may have residual respiratory abnormalities secondary to fungus-induced fibrosis.^11^^,^^12^ The present report describes a case with minimal clinical manifestations but with some CT findings that ultimately lead to the etiological diagnosis.

The chronic form of PCM can be classified as mild, moderate and severe. Severe cases are defined by meeting three or more of the following criteria:[13] I) Weight loss greater than 10% of usual weight; II) Intense pulmonary involvement; III) Involvement of other organs, such as adrenal glands, central nervous system and bones; IV) Presence of affected lymph nodes in multiple chains, superficial or deep, of the tumoral type (> 2.0 cm in diameter, without suppuration) or of the suppurative type; V) High antibody titers.^13^ Severe cases are represented by patients with clinical instability, due to respiratory failure, adrenal dysfunction, neurological syndrome or acute abdomen.

Mild cases are those with weight loss below 5% of usual weight and involvement of a single organ or slight involvement of organs or tissues without dysfunction.^13^

The severity criteria described above are useful in the diagnostic elaboration, as well as in the therapeutic planning. Dual Immunodiffusion (IDD), Counter Immunoelectrophoresis (CIE), Enzyme-Linked Immunosorbent Assay (ELISA) and immunoblot methods are currently available in different reference services.^13^

Using standardized techniques and appropriate antigens, these tests have a sensitivity between 80% and 95%. The titer of specific anti-*P. brasiliensis* is correlated with severity by *P. lutzii.* The specificity of serological tests varies from 85% to 100%, with immunodiffusion being considered the most specific.^2^^,^^4^^,^^12^ False-positive reactions may occur with sera from patients with histoplasmosis and, eventually, in aspergillosis. In view of the greater simplicity of the test, sensitivity > 80% and specificity > 90%, as well as the experience accumulated in recent decades, the double immunodiffusion reaction in agar gel is currently the main method of serological diagnosis of PCM.^4^ Recently, molecular detection of *Paracoccidioides* spp. And dot blotting assays have been proposed for diagnosis of PCM, although these diagnostic tools are still not available for clinical use.^14^, ^15^, ^16^

It is recommended for IDD or any other test used in the diagnosis of PCM that the sera be titrated for a better interpretation of the therapeutic response, since the antibody titers progressively decrease with the clinical control of the disease. It is desirable that negative or stabilization occur in a dilution of 1:2 or less to consider the criterion of serological cure fulfilled.^4^^,^^13^ Some patients may have titers below 1:4 at diagnosis. In these cases, the serological criterion by the IDD has limited value in the follow-up of PCM care treatment.^13^ Treatment of mild and moderate forms of PCM is based on the use of Itraconazole, at a daily dose of 200 mg for nine to 18 months with an average of 12 months, with high rates of efficacy and safety, although cotrimoxazole, amphotericin B and other azoles can be used as alternatives.^16^ In the present case, the patient received itraconazole and presented with regression of lungs lesions without evidence of sequelae.

Our report indicates that PCM should be considered as a potential diagnosis in patients with no conventional risk factors, presenting a clinical picture that could resemble the disease, even in the absence of suggestive epidemiological findings.

## Conflicts of interest

The authors declare that the research was conducted in the absence of any commercial or financial relationships that could be construed as a potential conflict of interest.
